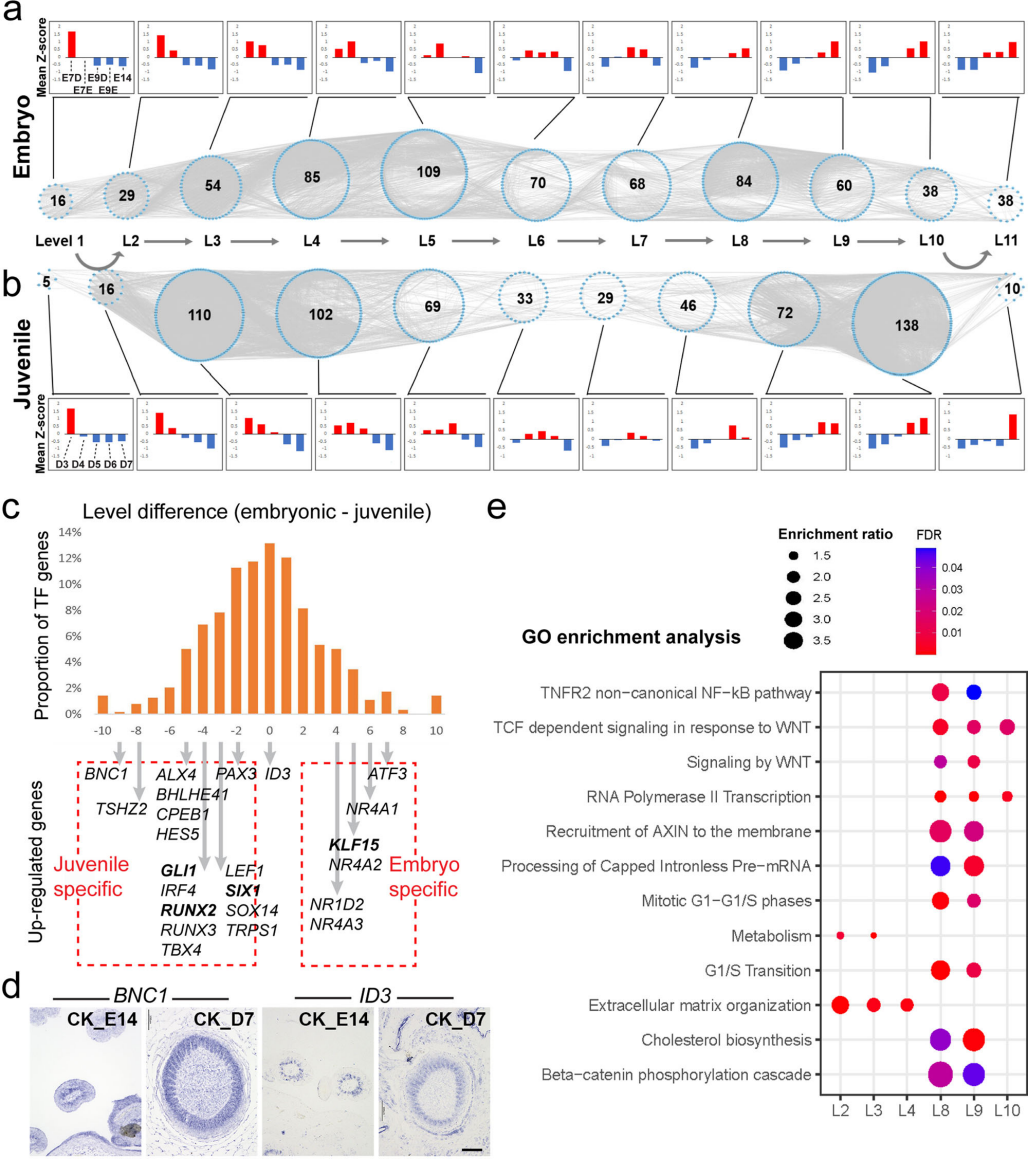# Author Correction: Conserved regulatory switches for the transition from natal down to juvenile feather in birds

**DOI:** 10.1038/s41467-024-49682-3

**Published:** 2024-06-25

**Authors:** Chih-Kuan Chen, Yao-Ming Chang, Ting-Xin Jiang, ZhiCao Yue, Tzu-Yu Liu, Jiayi Lu, Zhou Yu, Jinn-Jy Lin, Trieu-Duc Vu, Tao-Yu Huang, Hans I-Chen Harn, Chen Siang Ng, Ping Wu, Cheng-Ming Chuong, Wen‐Hsiung Li

**Affiliations:** 1 Department of Pathology, Keck School of Medicine, University of Southern California, Los Angeles, CA USA; 2 Biodiversity Research Center, Academia Sinica, Taipei, Taiwan; 3 The iEGG and Animal Biotechnology Center, National Chung Hsing University, Taichung, Taiwan; 4 Rong Hsing Research Center for Translational Medicine, National Chung Hsing University, Taichung, Taiwan; 5 Institute of Biomedical Sciences, Academia Sinica, Taipei, Taiwan; 6 Department of Cell Biology and Medical Genetics, Shenzhen University Medical School, Shenzhen, Guangdong China; 7 International Cancer Center, Shenzhen University Medical School, Shenzhen, Guangdong China; 8 Guangdong Key Laboratory of Genome Instability and Human Disease Prevention, Shenzhen University Medical School, Shenzhen, Guangdong China; 9 Department of Life Sciences, National Cheng Kung University, Tainan, Taiwan; 10 National Applied Research Laboratories, National Center for High-performance Computing, Hsinchu, Taiwan; 11 Michigan Neuroscience Institute, University of Michigan School of Medicine, Ann Arbor, MI USA; 12 Institute of Molecular and Cellular Biology, National Tsing Hua University, Hsinchu, Taiwan; 13 Department of Life Science, National Tsing Hua University, Hsinchu, Taiwan; 14 Bioresource Conservation Research Center, National Tsing Hua University, Hsinchu, Taiwan; 15 Department of Ecology and Evolution, University of Chicago, Chicago, IL USA

**Keywords:** Evolutionary developmental biology, Gene regulatory networks, Differentiation

Correction to: *Nature Communications* 10.1038/s41467-024-48303-3, published online 16 May 2024

The original version of this Article contained errors in Fig. 2a. Firstly, the sample order in the histogram was erroneously given as “E7D, E9D, E7E, E9E, E14” rather than “E7D, E7E, E9D, E9E, E14” as intended. Secondly, a letter “e” was inadvertently superimposed on the top of the label “L9”. These errors have been corrected in both the PDF and HTML versions of the Article.