# Supplementary material for: Conserved regulatory switches for the transition from natal down to juvenile feather in birds
Source: Nat Commun. 2024 May 16;15:4174. doi: 10.1038/s41467-024-48303-3 (PMC11099144; doi:10.1038/s41467-024-48303-3)
Supplement: Supplementary file 1 — Supplementary Information [file 41467_2024_48303_MOESM1_ESM.pdf]

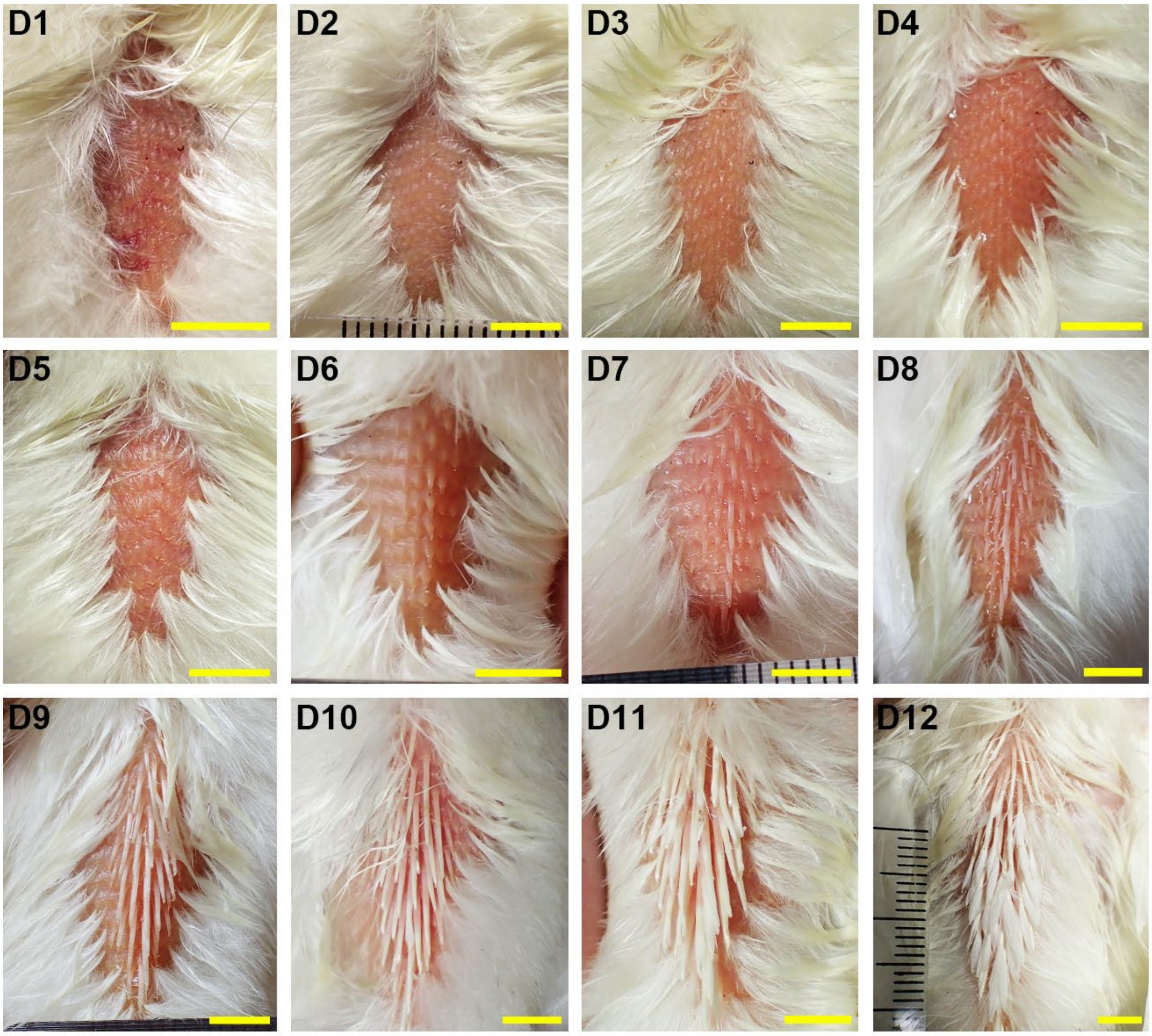

Supplementary Figure 1. Dorsal views of developing chicken juvenile feathers. The posterior dorsal natal downs were removed to visualize the beneath juvenile feather germs. D: posthatch days. Scale bar: 1 cm.

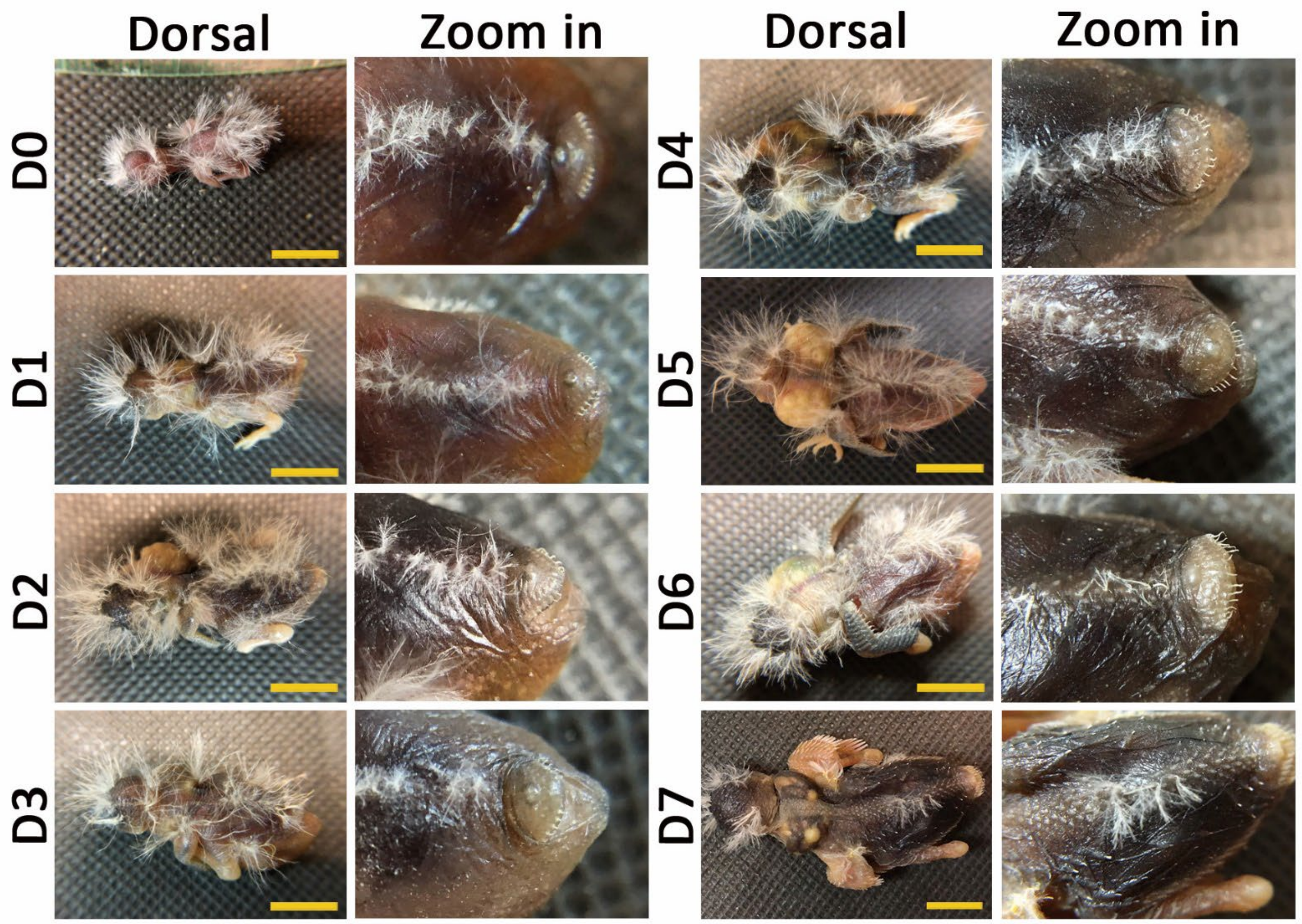

Supplementary Figure 2. Dorsal views and the enlargements of developing zebra finch juvenile feathers. The natal downs in the enlargement views were trimmed to visualize the beneath juvenile feather germs. D: posthatch days. Scale bar: 1 cm.

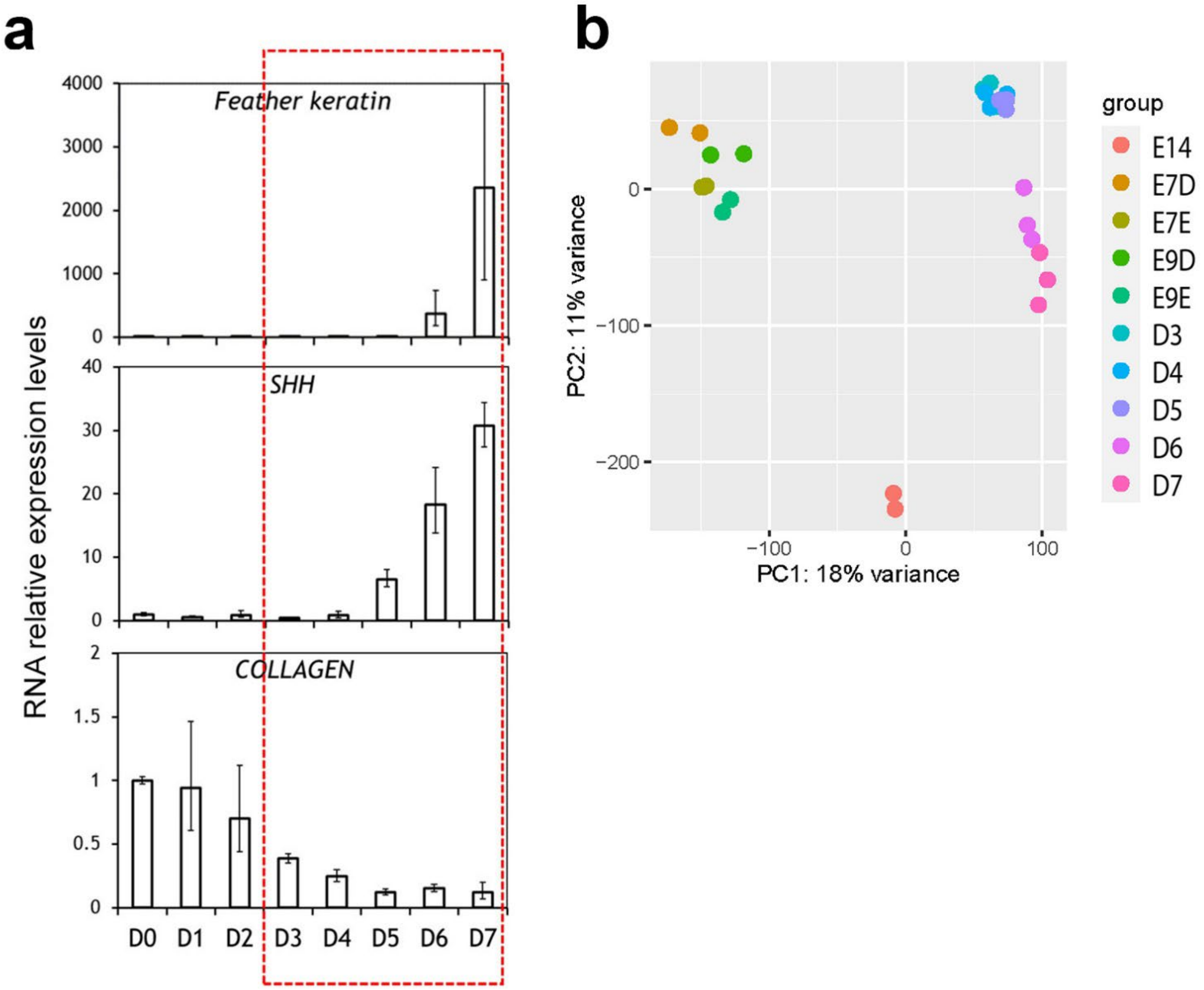

Supplementary Figure 3. Quality controls of the samples in transcriptomic analysis. a Quantitative PCR detection of sevral morphogems at different developmental stages of chicken dorsal skins. (*n* = 3 biologically independent samples). The error bar is given as SD. The raw data are shown in Source Data file. **b** PCA analysis of all the RNA-seq libraries used in this study. E14: E14 feather filament; E7D: E7 dermis; E7E: E7 epidermis; E9D: E9 dermis; E9E: E9 epidermis; D3, 4, 5, 6, 7: posthatch day 3, 4, 5, 6, 7, respectively.

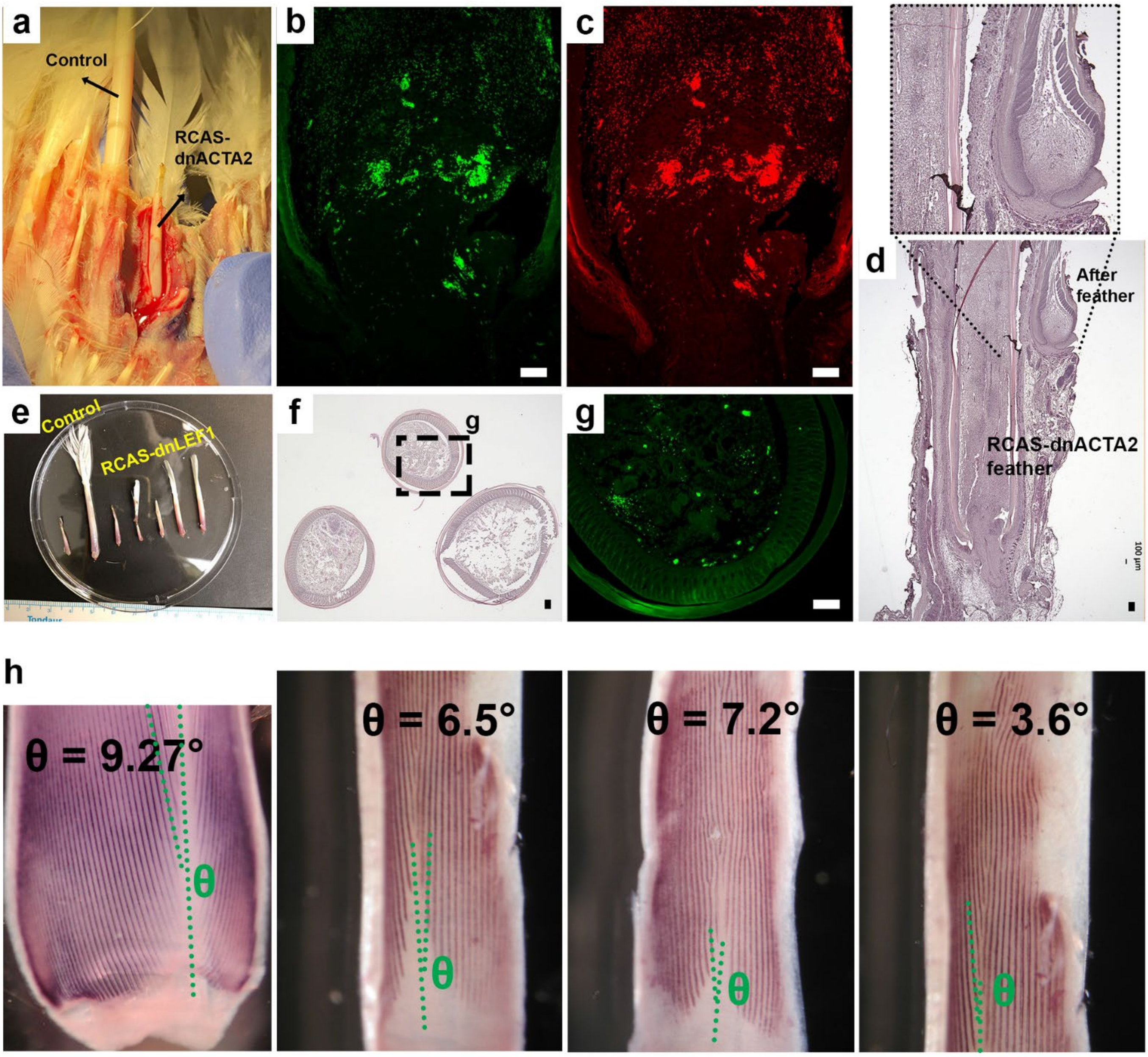

Supplementary Figure 4. Supplementary figures for ACTA2 and LEF1 functional analysis. a The dissected flight feather follicles with or without RCAS-dnACTA2 treatment. **b** and **c** IHC with AMV3C2 (e) and LCAM (f) of the longitudinal sections of a regenerated flight feather follicle with RCAS-dnACTA2 injection. **d** H&E staining of dnACTA2 overexpressed follicle and the adjescent after feather (n = 5 biologically independent samples). The dnACTA2 overexpressed follicle can not be renewed and lost the barb structures. The after feather follicle on the up right side showing normal barb structure is used for the comparison. **e** The dissected flight feather follicles with or without RCAS-dnLEF1 treatment. **f** H&E staining of the three cross sections of flight feather follicles with RCAS-dnLEF1 injection. The top one lost the entire rachis while the bottom two showed abnormal small rachis. **g** The virus staining (AMV3C2) of the squre in f. **h** Shh *in situ* to show the marginal plate that highlight the barb ridges. The measurements of θ values in control (the most left pane) and different dnLEF1 overexpressed follicles are shown.

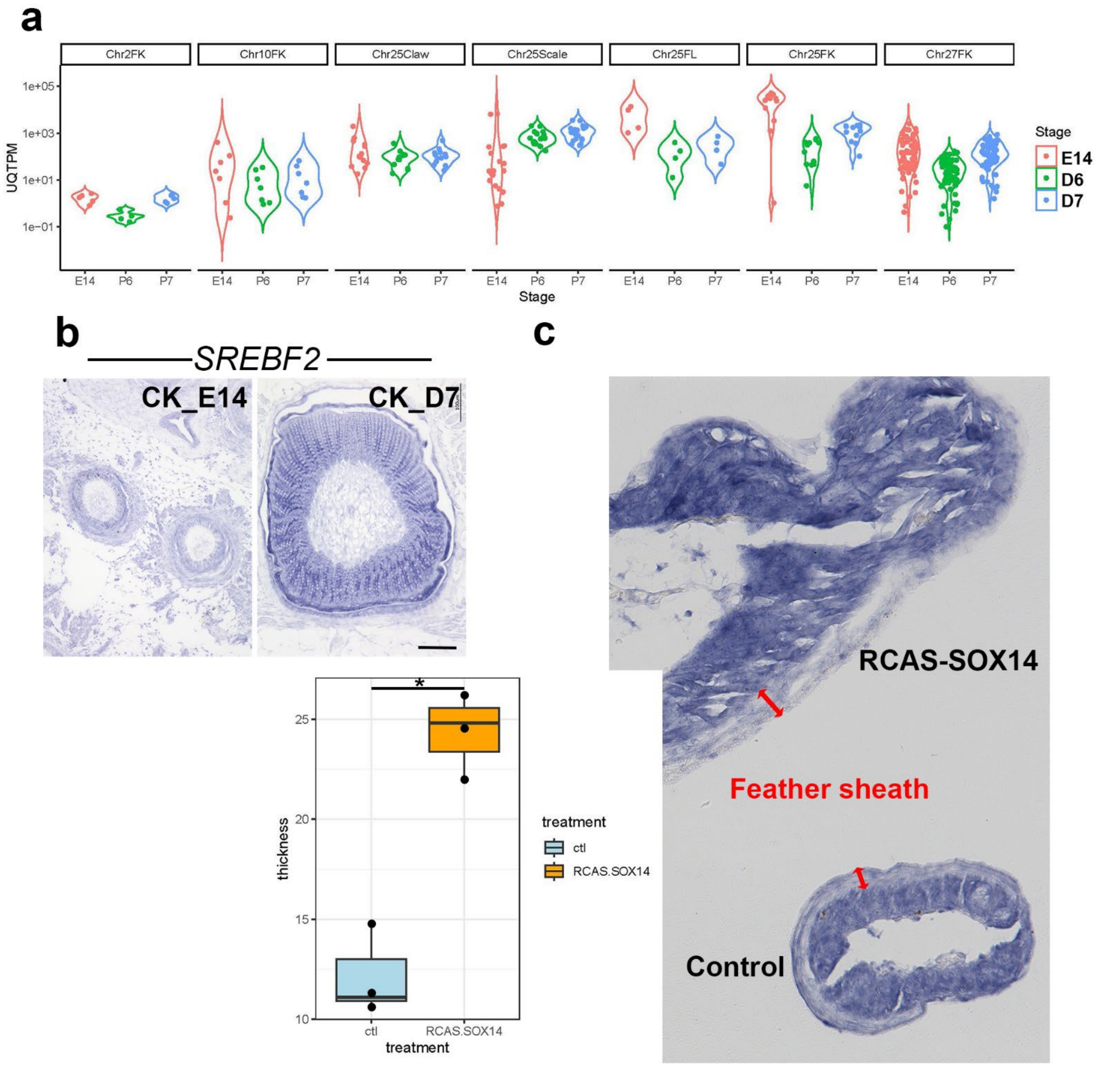

Supplementary Figure 5. Supplementary figures for feather sheath analysis. a Violin plots of the expression levels of β-keratin gene in different β-keratin subfamilies. The raw data are shown in Source Data file. **b** SISH of *SREBF2* in cross sections of E14 (CK_E14) and D7 chicken (CK_D7) feather follicles. (n = 5 biologically independent samples). **c** the enlargement of RCAS-SOX14 misexpressed skin (Fig. 5l, SISH of *SOX14*) shows the virus-infected and adjescent control follicles with higher and lower *SOX14* expression, respectively. The feather sheaths are indicated by red arrows and the thicknesses were compared between 3 virus-infected and 3 control follicles (p-value = 0.00138, one-sided Student's t-test. Unit: µm). Interquartile range (IQR) of boxplot is between Q1 and Q3 and centre line indicates median value. Whiskers of boxplot is extended to the maxima and minima. Maxima is Q3 + 1.5*IQR and minima is Q1 - 1.5*IQR. The raw data are shown in Source Data file. UQTPM: upper quartile TPM values. FK: feather keratin; Claw: claw keratin; Scale: scale keratin; FL: feather keratin like protein; E14: E14 feather filament; D6: posthatch day 6; D7: posthatch day 7. Scale bar: 100 µm.

Supplementary Table 1. IDs and descriptions of the transcriptomic libraries.

Library ID	Description	Source	Replicates
E7E	Embryonic day 7 epidermis	Made in this study	2
E7D	Embryonic day 7 dermis	Made in this study	2
E9E	Embryonic day 9 epidermis	Made in this study	2
E9D	Embryonic day 9 dermis	Made in this study	2
E12F	Embryonic day 12 feather filaments	From [1, 2]	2
E14F	Embryonic day 14 feather filaments	Made in this study	2
E16F	Embryonic day 16 feather filaments	From [1, 2]	2
D3	Posthatch day 3 skin	Made in this study	3
D4	Posthatch day 4 skin	Made in this study	3
D5	Posthatch day 5 skin	Made in this study	3
D6	Posthatch day 6 skin	Made in this study	3
D7	Posthatch day 7 skin	Made in this study	3

References

1. Lin GW, Lai YC, Liang YC, Widelitz RB, Wu P, Chuong CM. Regional Specific Differentiation of Integumentary Organs: Regulation of Gene Clusters within the Avian Epidermal Differentiation Complex and Impacts of SATB2 Overexpression. *Genes (Basel)* **12**, (2021).

2. Liang YC*, et al.* Folding Keratin Gene Clusters during Skin Regional Specification. *Dev Cell* **53**, 561-576 e569 (2020).
