# Supplementary material for: Conserved regulatory switches for the transition from natal down to juvenile feather in birds
Source: Nat Commun. 2024 May 16;15:4174. doi: 10.1038/s41467-024-48303-3 (PMC11099144; doi:10.1038/s41467-024-48303-3)
Supplement: Supplementary file 3 — Description of Additional Supplementary Files [file 41467_2024_48303_MOESM3_ESM.pdf]

Description of Additional Supplementary Files:

Supplementary Data 1: TPM_table_all_samples. A TPM datasheet includes the sample descriptions and TPM values of genes across samples.

Supplementary Data 2: differencial_TOGCN_level_bw_embryonic_and_posthatch_and_DEGs. A datasheet contains differential TO-GCN levels between embryonic and posthatch tissues as well as the DEGs from different comparisons.

Supplementary Data 3: Enriched_Reactome_pathways_for_embryonic_samples. A data sheet with the enriched reactome pathways in embryonic TO-GCN.

Supplementary Data 4: Enriched_Reactome_pathways_for_posthatch_samples. A data sheet with the enriched reactome pathways in posthatch TO-GCN.

Supplementary Data 5: TO-GCN_ref_overlapped_TFs_level_8to10_diff3. A datasheet shows the overlapping between TFs with higher than 3 level differences in level 8 to 10 of TO-GCN and two published researches.

Supplementary Data 6: TF_coexpressed_genes_with_TO-GCN_level. A datasheet shows the genes that are co-expressed with the TFs (with TO-GCN level).

Supplementary Data 7: TPMmt1unique_coding_keratinocyte_AVG_stringtie_- e_gg6_0129_FT. A TPM table shows the expressions of all the manually annotated keratin genes across tissues.
