# Supplementary material for: Conserved regulatory switches for the transition from natal down to juvenile feather in birds
Source: Nat Commun. 2024 May 16;15:4174. doi: 10.1038/s41467-024-48303-3 (PMC11099144; doi:10.1038/s41467-024-48303-3)
Supplement: Supplementary file 11 — Reporting Summary [file 41467_2024_48303_MOESM11_ESM.pdf]

nature portfolio

Corresponding author(s): Chih-Kuan Chen
Cheng-Ming Chuong
Wen-Hsiung Li

Last updated by author(s): Mar 6, 2024

Reporting Summary

Nature Portfolio wishes to improve the reproducibility of the work that we publish. This form provides structure for consistency and transparency in reporting. For further information on Nature Portfolio policies, see our Editorial Policies and the Editorial Policy Checklist.

Statistics

For all statistical analyses, confirm that the following items are present in the figure legend, table legend, main text, or Methods section.

n/a	Confirmed	
☐	☒	The exact sample size (*n*) for each experimental group/condition, given as a discrete number and unit of measurement
☐	☒	A statement on whether measurements were taken from distinct samples or whether the same sample was measured repeatedly
☐	☒	The statistical test(s) used AND whether they are one- or two-sided<br>*Only common tests should be described solely by name; describe more complex techniques in the Methods section.*
☒	☐	A description of all covariates tested
☒	☐	A description of any assumptions or corrections, such as tests of normality and adjustment for multiple comparisons
☐	☒	A full description of the statistical parameters including central tendency (e.g. means) or other basic estimates (e.g. regression coefficient) AND variation (e.g. standard deviation) or associated estimates of uncertainty (e.g. confidence intervals)
☐	☒	For null hypothesis testing, the test statistic (e.g. *F*, *t*, *r*) with confidence intervals, effect sizes, degrees of freedom and *P* value noted<br>*Give P values as exact values whenever suitable.*
☒	☐	For Bayesian analysis, information on the choice of priors and Markov chain Monte Carlo settings
☐	☒	For hierarchical and complex designs, identification of the appropriate level for tests and full reporting of outcomes
☒	☐	Estimates of effect sizes (e.g. Cohen's *d*, Pearson's *r*), indicating how they were calculated

Our web collection on statistics for biologists contains articles on many of the points above.

Software and code

Policy information about availability of computer code

Data collection	no software was used to collect data in this study
Data analysis	RNA-seq analysis: Trimmomatic_v0.36 for reads trimming; Hisat2/2.1.0 for reads mapping (reference genomes of GRCg7w for TO-GCN and GRCg6a for keratin annotation); StringTie /1.3.4d for assembling the transcripts; The R package DESeq2/1.36.0 for differential expression analysis. ATAC-seq analysis: Cutadapt/3.4 for reads trimming; Hisat2/2.1.0 for reads mapping (to GRCg6a); Samtools /1.10 for low quality and non-unique hits removal; Picard/2.26.2 toolkit for PCR duplicates removal; MACS2/ 2.2.7.1 for peak calling; HINT-ATAC for footprint analysis. The codes used for TO-GCN analyses are available at https://github.com/petitmingchang/TO-GCN. The code used for ATAC peak visualization is available at https://github.com/r93b42016/feather_transition.

For manuscripts utilizing custom algorithms or software that are central to the research but not yet described in published literature, software must be made available to editors and reviewers. We strongly encourage code deposition in a community repository (e.g. GitHub). See the Nature Portfolio guidelines for submitting code & software for further information.

Data

Policy information about availability of data

All manuscripts must include a data availability statement. This statement should provide the following information, where applicable:

- Accession codes, unique identifiers, or web links for publicly available datasets
- A description of any restrictions on data availability
- For clinical datasets or third party data, please ensure that the statement adheres to our policy

High throughput sequencing data have been deposited in the NCBI Sequence Read Archive (SRA) under BioProject ID: PRJNA1084783

Research involving human participants, their data, or biological material

Policy information about studies with human participants or human data. See also policy information about sex, gender (identity/presentation), and sexual orientation and race, ethnicity and racism.

Reporting on sex and gender	no human data/sample was involved.
Reporting on race, ethnicity, or other socially relevant groupings	no human data/sample was involved.
Population characteristics	no human data/sample was involved.
Recruitment	no human data/sample was involved.
Ethics oversight	no human data/sample was involved.

Note that full information on the approval of the study protocol must also be provided in the manuscript.

Field-specific reporting

Please select the one below that is the best fit for your research. If you are not sure, read the appropriate sections before making your selection.

- [x] Life sciences
- [ ] Behavioural & social sciences
- [ ] Ecological, evolutionary & environmental sciences

For a reference copy of the document with all sections, see nature.com/documents/nr-reporting-summary-flat.pdf

Life sciences study design

All studies must disclose on these points even when the disclosure is negative.

Sample size	We have calculated sample size based on a 95% confidence level to determine the minimal number of sample size necessary to obtain sufficient statistical power. The number is determined to be triplicates for RT-PCR, two or three replicates for RNA-seq, and triplicates for ATAC-seq. At least 5 replicates for each functional validation (for both control and gene misexpression).
Data exclusions	No data was excluded.
Replication	At least 2 independent rounds of 3 chickens per control/experimental group were carried out to achieve the final sample size higher than 6 in this study. Independent duplicates were used for RT-PCR, RNA-seq, or ATAC-seq.
Randomization	Animals were randomly selected.
Blinding	Blinding was applied during data quantification and interpretation of morphological changes.

Reporting for specific materials, systems and methods

We require information from authors about some types of materials, experimental systems and methods used in many studies. Here, indicate whether each material, system or method listed is relevant to your study. If you are not sure if a list item applies to your research, read the appropriate section before selecting a response.

Materials & experimental systems

n/a	Involved in the study	
☐	☒	Antibodies
☒	☐	Eukaryotic cell lines
☒	☐	Palaeontology and archaeology
☐	☒	Animals and other organisms
☒	☐	Clinical data
☒	☐	Dual use research of concern
☒	☐	Plants

Methods

n/a	Involved in the study	
☒	☐	ChIP-seq
☒	☐	Flow cytometry
☒	☐	MRI-based neuroimaging

Antibodies

Antibodies used	α-SMA (Invitrogen, MA1-06110, 1:50), vimentin (Developmental Studies Hybridoma Bank, H5, 1:30)), ZEB2 (Proteintech, 14026-1-AP, 1:50). NCAM and TNC were from the Chuong lab (1:100)
Validation	α-SMA (Invitrogen, MA1-06110, 1:50): validated to interact with chicken IHC in this study.<br>vimentin (Developmental Studies Hybridoma Bank, H5, 1:30): validated to interact with chicken IHC by the manufacturer. Applied in chicken IHC by Baeriswyl, T. and Stoeckli, E. T. Neural Development (2008, PMID: 18346270).<br>ZEB2 (Proteintech, 14026-1-AP, 1:50): validated to interact with chicken IHC in this study.<br>NCAM and TNC were from the Chuong lab (1:100): Applied in chicken IHC by Wu, P. et al. Development (2021, PMID: 34344024).

Animals and other research organisms

Policy information about studies involving animals; ARRIVE guidelines recommended for reporting animal research, and Sex and Gender in Research

Laboratory animals	White Leghorn chicken (Gallus gallus: E7, E9, E14, D3, D4, D5, D6, D7, 1-month-old). Both sexes were used.<br>Zebra finch (Taeniopygia guttata wild type: E12, D0, D4, D7). Both sexes were used.
Wild animals	no wild animals were involved.
Reporting on sex	no differences between sexes
Field-collected samples	no field-collected samples were involved
Ethics oversight	All the animals used in this study were processed following the approved protocols of the Institutional Animal Care and Use Committees of the University of Southern California (USC; Los Angeles, CA, USA) and Institutional Animal Care and Use Committees of National Chung Hsing University (NCHU, Taichung, Taiwan).

Note that full information on the approval of the study protocol must also be provided in the manuscript.

Plants

Seed stocks	*Report on the source of all seed stocks or other plant material used. If applicable, state the seed stock centre and catalogue number. If plant specimens were collected from the field, describe the collection location, date and sampling procedures.*
Novel plant genotypes	*Describe the methods by which all novel plant genotypes were produced. This includes those generated by transgenic approaches, gene editing, chemical/radiation-based mutagenesis and hybridization. For transgenic lines, describe the transformation method, the number of independent lines analyzed and the generation upon which experiments were performed. For gene-edited lines, describe the editor used, the endogenous sequence targeted for editing, the targeting guide RNA sequence (if applicable) and how the editor was applied.*
Authentication	*Describe any authentication procedures for each seed stock used or novel genotype generated. Describe any experiments used to assess the effect of a mutation and, where applicable, how potential secondary effects (e.g. second site T-DNA insertions, mosiacism, off-target gene editing) were examined.*
